# Effects of Free Play and Partly Structured Playground Activity on Motor Competence in Preschool Children: A Pragmatic Comparison Trial

**DOI:** 10.3390/ijerph19137652

**Published:** 2022-06-22

**Authors:** Patrizia Tortella, Monika Haga, Håvard Lorås, Guido Francesco Fumagalli, Hermundur Sigmundsson

**Affiliations:** 1 Faculty of Human and Social Sciences, University of Enna “Kore”, 94100 Enna, Italy; patrizia.tortella@gmail.com; 2 Associazione Onlus Laboratorio 0246, Via del Nascimben, 1b, 31100 Treviso, Italy; guido.fumagalli50@gmail.com; 3 Department of Teacher Education, Section for Arts, Physical Education and Sports, Norwegian University of Science and Technology (NTNU), 7010 Trondheim, Norway; havard.loras@ntnu.no; 4 Public Health and Community Medicine, Research Center on Child Motor Development, University of Verona, 37100 Verona, Italy; 5 Department of Psychology, Norwegian University of Science and Technology, 7010 Trondheim, Norway; hermundur.sigmundsson@ntnu.no; 6 Research Center for Education and Mindset, University of Iceland, 101 Reykjavik, Iceland

**Keywords:** motor skill, scaffolding, playground, active play, deliberate preparation

## Abstract

Both the indoor and the outdoor environments and their organization exert pronounced influence upon physical activity behavior and motor development of preschool children. The aim of this study was to explore whether partly structured activity or free play in a specific playground had different impacts on motor competence development in 4–6-year-old preschoolers. The study had a pretest–post-test design, with two intervention groups and one control. Sixty-two children were included in a partly structured activity group and forty-three children in a free-play group. Both groups participated in playground activities consisting of 10 sessions (once a week), each lasting 1 h, in a specific playground setting. For the partly structured activity group, activities in each session consisted of a combination of both structured and free activity while the free-play group were engaged in unstructured play only. The control group did not attend the playground activities (N = 36). To assess levels of motor skills, each child completed pre- and post-tests using the Movement Assessment Battery for children (MABC-2), the Test of Motor Competence (TMC) and two playground-specific tests. A 3 (study group) and X 2 (gender) ANCOVAs were conducted on post-test scores on each of the test items from TMC, MABC-II and playground tests, with pre-test scores as covariates. Post hoc pairwise multiple comparisons were conducted with the alpha Bonferroni corrected, and the partial eta-squared (*η*^2^*_p_*) was applied as a measure of effect size. The results indicate no significant differences in motor competence measured by the TMC or the MABC-2 between groups. On the contrary, a significant improvement in performance in the playground-specific tests was observed in the partly structured activity group compared to the free-play and control groups.

## 1. Introduction

Data produced in recent years indicate a negative trend in children’s motor competence [1,2,3]. This may be linked to the findings that many young children do not meet the daily physical activity time recommendations, with reduced levels of physical activity and increased time engaging in sedentary behavior [4,5]. Motor behavior is a fundamental component in everyday life; performing precise and coordinated movements adapted to the environment is fundamental to a child’s participation and function in various activities such as play, locomotion and dressing [6,7]. In this respect, the term motor competence has been postulated to conceptualize a person’s level of performance when executing different motor acts [6,7]. The term incorporates both fine motor skills/activities, the coordination of small muscle movements such as those of the fingers, and gross motor skills/activities, which involve the coordinated participation of large muscle groups, and whole-body movements.

A positive association is found between motor competence, participation in physical activities and physical fitness components in young people [8,9]. There are also positive associations between self-perception [10], healthy identity development [11] and resilience [12]. Apparently, spending time in physical activities will influence the development of motor competence and physical fitness, and vice versa; thus, individuals who master different movements and have a comprehensive motor repertoire tend to have an improved basis for participation in various physical activity play, sports and games, and consequently increase their fitness levels [13]. Stodden et al. [14] propose that the development of motor skill competence is a fundamental underlying mechanism promoting engagement in physical activity. In the conceptual model, the authors hypothesize a strong relationship among motor skill competence, physical activity, perceived motor skill competence, health-related physical fitness and obesity. Acquisition of motor competences should therefore be encouraged in children, as it may support positive and sustainable trajectories of health behavior and lead to long-term health outcomes [15].

Active, or free, play is defined as “a form of gross motor or total body movement in which young children exert energy in a freely chosen, fun, and unstructured manner” [16], p. 164. Although free play is important for versatile movement experience, it can be argued that this is not enough to achieve motor learning levels that promote lifelong joy of movement and participation in physical activity [17,18]. It has been proposed that to create a robust base of motor competence and motor confidence, the learning process of motor skills (i.e., locomotion, balance and manipulative skills) should be facilitated through appropriate feedback, instruction and organization (McNamara et al., 2015). Giblin et al. [19] refer to this approach as “Deliberate Preparation”, underlining that children must be supported, guided and encouraged through a range of developmentally appropriate tasks during the motor learning process. This is reflected in the physical activity guidelines for physical activity for children under 5 years of age, as they include recommendations for a combination of structured activity and free play [20]. 

Motor development and learning is a dynamic process characterized by continuous interactions between the different constraints in the child, the task and the environment [21]. Young children develop and learn new motor skills in a body that is continually changing within an environment that generates as well as limits possibilities for action [22]. Both theories and empirical findings on motor learning suggest that the time spent engaging in a task, such as duration and frequency, and task salience are good predictors of progression in motor skill development [23,24,25]. Accordingly, learning in the course of development may be quite specific, and specific training may affect the development of some motor skill competences and not others [26,27]. 

There is evidence suggesting that structured programs are effective in improving and maintaining fine and gross motor skills [28,29,30,31,32,33]. Moreover, structured activity appears to be more efficient than free play in producing high physical engagement during activity in preschool children [34,35,36]. However, results indicate that limited transfer occurred between tasks referring to different domains of motor competences, i.e., practicing with a specific tool has a direct impact on that task only and not necessarily on other tasks related to the same competence [24,31]. In sum, due to the small number of eligible studies, no conclusion on the effect of different modalities of play interventions on preschoolers’ physical activity levels and motor skills can be drawn.

A broad range of factors are found to be potential influencers for motor learning and development, including cultural, biological, environmental, social and psychological factors [37]. Moreover, both the indoor and the outdoor environments (e.g., equipment, organization, urban/rural areas) and their organization exert pronounced influences upon the physical activity behavior and motor development of preschool children [38,39]. In this context, the issue of how the organization, structure and purpose of the movement activities and active play may influence motor development in preschoolers has so far received limited attention [2,33,40]. Accordingly, the aim of this study was to determine whether partly structured activity or free play in a specific playground had different impacts on motor competence development in 4–6-year-old preschoolers. 

## 2. Materials and Methods

### 2.1. Participants

The study had a pretest–post-test design, with two intervention groups and one control. Children were recruited from 5 kindergartens in the city of Treviso (Italy) with pupils of similar socio-economic status and ethnic origin. No child had any reported history of learning difficulties or any behavioral, neurological or orthopedic problem that would qualify as an exclusionary criterion for this study. One hundred and forty-one children aged 4 to 6 years old completed the measurement at pre- and post-test. A total of 62 children (28 boys and 34 girls; mean age 5.1 ± 0.6 years) from two kindergartens formed the partly structured activity group. A total of 43 children from two other kindergartens (18 boys and 25 girls, mean age 5.1 ± 0.6 years) formed the free-play group. The control group consisted of 36 children, 22 boys and 14 girls from another kindergarten who did not attend the playground activities (mean age 5.5 ± 0.4 years).

### 2.2. Procedures

The Scientific Committee of Laboratorio 0246 approved the data collection process and the entire study. The study was in line with the principles of the Declaration of Helsinki. Information about the study and a written consent form were sent to the parents or guardians. Field permission for the study was granted by: ASD Laboratorio 0246 no-profit—Strada del Nascimben 1/B 31100 Treviso, phone: +39-0422-324310, Fax: +39-0422-324311, email: info@0246.it; http://www.0246.it/. 

### 2.3. The Primo Sport 0246 Playground

**Figure 1:**
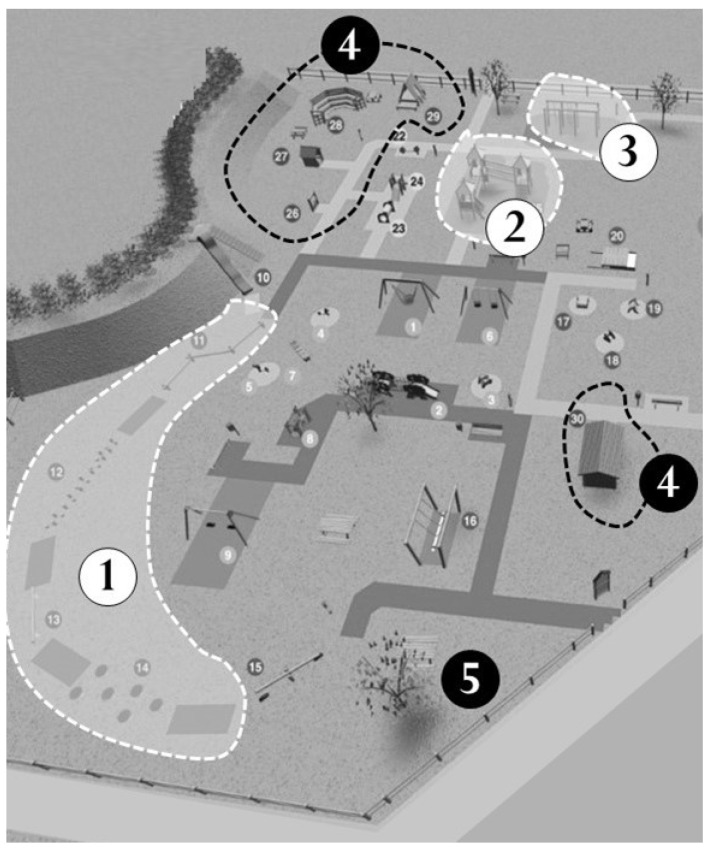
Layout of the playground Primo Sport 0246 and distribution of the specific areas. 1: balance area; 2: mobility area; 3: manual dexterity area; 4: symbolic play area; 5: mixed area.

The playground Primo Sport 0246 (Figure 1) in Treviso, Italy, is a privately funded playground open to the public and was designed to promote motor development in children from 0 to 6 years [41]. The playground extends over 2500 mq and contains a total of 35 playground equipment and instruments. The playground has five dedicated areas equipped with different instruments: balance area; mobility area; manual dexterity area; symbolic play area; and mixed area (Figure 1). In the manual dexterity, mobility and balance areas, there are instruments that could be used for children at different motor skill levels/levels of motor competence. (For a detailed description of the organization of the park, see [41]).

### 2.4. Organization of Activities at the Playground

For the purposes of this study, a structured activity session is defined as the time spent performing a planned combination of activities designed to incorporate opportunities to practice basic motor skills and make use of large muscle groups (Tortella et al., 2019). The sessions of all groups took place in springtime (March to May 2017) between 9 am and 12 am, with temperatures ranging between 15 and 26 degrees Celsius; no rainy days were experienced during the study. The two groups attended the playground on different days. The playground activities consisted of 10 sessions, each lasting 1 h, executed once per week. During each visit, the children of the partly structured activity group were exposed for 30 min to structured activity and for 30 min to free play; the other group of children participated in 60 min of free play only (free-play group). Both groups participated in the movement session in the same outdoor playground with the same facilities and equipment available. Instructors with degrees in exercise science were trained to conduct the activities at the park. Before the intervention period began, they attended 3 theoretical and 2 practical sessions in the park with children from a preschool outside the research. In the practical sessions, the instructors were trained to apply the protocol, individually supervised by one of the authors. During the sessions, the instructor’s role was to guide and scaffold the structured activities. They did not participate in children’s activities as a “playmate” either during the structured activity or during free play.

#### 2.4.1. The Group of Partly Structured Activity

**Figure 2:**
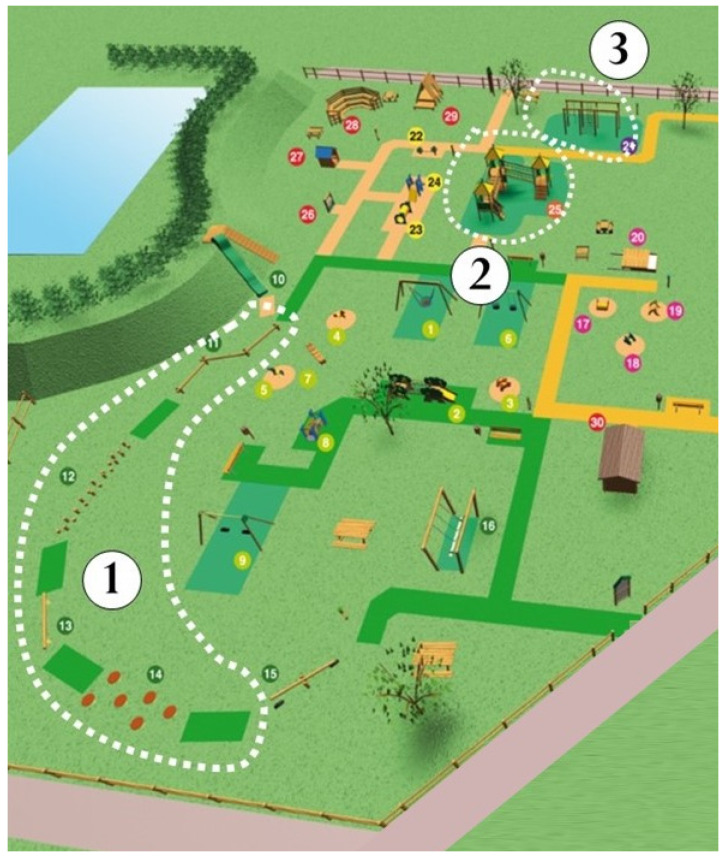
Dotted lines limit the areas where structured activities were run. 1: Balance area; 2: mobility area; 3: manual dexterity area. Active play (unstructured activities) could be performed everywhere else in the park according to children’s choice.

**Figure 3:**
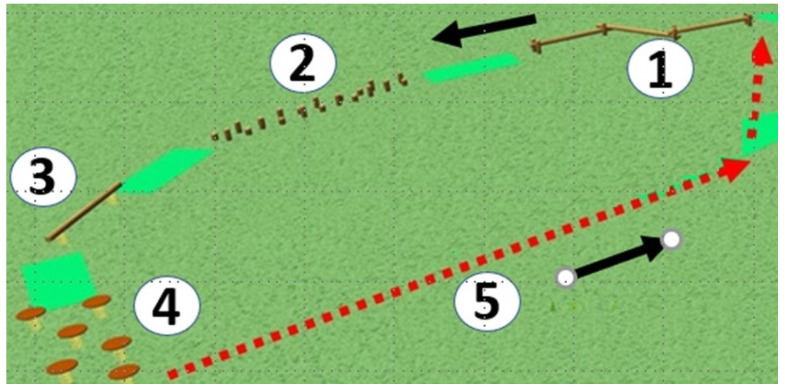
Motor circuit in the balance area. The children started the balance path from point 1 and continued in succession to point 5 and then started again. They stayed in the balance area for 10 min, completing on average 2–4 full trails.

The group was divided into two subgroups of the same size. One started with free play while the other was involved in the structured activity; after 30 min, the groups switched type of activity, e.g., from structured activity to free play or vice versa. When performing the structured activity, the subgroup was further divided into three small groups of 6–7 children with each small group spending 10 min in each of three dedicated areas (Figure 2). The sequence of the activities was specific for each dedicated areas: (1) manual dexterity: the children used the following equipment: rope ladder, climbing rope, hanging bar, gymnastic rings, climbing net, monkey bars; they stayed on a piece of equipment for about 30 s before moving to the next, spending a total of 10 min in this area. (2) Balance: the children played on the following fixed equipment in the sequence: balance beam, balance logs, elastic balance beam, elastic balance platforms (there was no loose equipment) (Figure 3). The children repeated the sequence about three times. (3) Mobility: each child went up and down from various climbing points and slopes in an organized sequence. During the 30 min section dedicated to free play, children could move everywhere within the playground, except for the portions of the areas that were occupied by the companion subgroup performing the structured activities. For each area, one trained instructor was constantly present. The instructors provided scaffolding if requested, gave instructions about possible ways to use instruments and provided general encouragement. An additional operator was present in each area to control the time children spent engaging in activities and coordinate the switch of the groups from one area to the other. Preschool teachers (at least one for every 10 children) were present for assistance and supervision in case of emergency; they were not involved in the activities (no instructions, guidance, or encouragement).

#### 2.4.2. The Group of Free Play

The group that engaged in free play could play freely everywhere on the playground for 60 min. An instructor was present at the playground to check the time. Preschool teachers were present for assistance and in case of emergency but were not involved in the activities.

#### 2.4.3. The Control Group

Children in the control group came to the playground only for the pre- and post-test. The control group carried out normal kindergarten activities as usual between the test times. All participating groups had access to outdoor space with some equipment in their kindergarten.

### 2.5. Assessment of Motor Competence

Assessment of motor competence was completed at the beginning and at the end of the study, i.e., after the groups had experienced the ten sessions at the playground. Test of Motor Competence (TMC) [42], MABC-2 [7] and two playground-specific tests (balance on beam and balance on elastic platform) [31] were used.

#### 2.5.1. Test of Motor Competence (TMC)

The TMC battery consists of four different tests: two fine motor tasks based on manual dexterity and two gross motor tasks based on dynamic balance. In all tasks, the performance measure was time to completion in seconds. The participants were given a practice run for each task. The TMC has an acceptable internal consistency of the test battery items, as all individual test item scores correlated positively with the total score with correlations ranging from 0.48 to 0.64. TMC has a construct validity of 0.47 for the Movement Assessment Battery for Children for 7-to 8-year-old children (n = 70) and a test–retest coefficient for the total score of 0.87 [42]. Quantification of aspects of fine motor performance was carried out with the brick handling tasks placing bricks (PB) and building bricks (BB). Two tests were used to quantify aspects of gross motor performance: heel-to-toe walking and walking/running in slopes.

*1. Placing bricks*. Eighteen square-shaped Duplo™ bricks were placed on a Duplo™ board (which has room for 3 × 6 bricks) as fast as possible. The participant was seated at a table and was given a practice run before the actual testing. The bricks had to be positioned in horizontal rows of three on the side of the active hand while the board was held firmly with the other hand. Both hands were tested.

*2. Building bricks*. Twelve square-shaped Duplo™ bricks were used to build a “tower” as fast as possible. The participant held one brick in one hand, and one brick in the other. At a signal, the participant assembled the bricks together one after one until all twelve had been put together to form a tower. Neither of the arms could rest on the table. The assembled bricks had to be held in the air the entire time. The tasks were conducted with participants sitting comfortably at a table, and time was stopped when the participants released the last brick. Brick handling has been used extensively in previous test batteries for motor performance [43]. 

*3. Heel-to-toe walking.* This task was adapted from the tandem walking test [44,45] and is a measure of dynamic balance capabilities. Participants were required to walk down a straight line (4.5 m long) marked on the floor as fast as they could while placing their heel against the toes of the foot in each step.

*4. Walking/running in slopes.* This task was an adaptation of the figure of eight test [46]. The participant started at the starting point. At a signal, the participant walked/ran as fast as possible in a figure of eight around two marked lines (1 m in width). Line 1 was 1 m from the starting point, and Line 2 was 5.5 m from the starting point. If the participant started to go on the right side of Line 1, the subject would go to the left side of Line 2, turn around and go back on the right side of Line 2 and left side of Line 1, and over the starting point. The time was stopped when the participant arrived at the starting point. Participants freely chose which direction they walked/ran. The participants wore suitable shoes.

#### 2.5.2. Movement Assessment Battery for Children (MABC-2)

The MABC-2 measures fine and gross motor coordination and is considered appropriate for ages 3–16 years [7]. The test contains diverse tasks for children at different age spans; we used the set for 3–6-year-old children. MABC-2 consists of 8 sub tests, allocated into 3 groups: (1) manual dexterity (posting coins, threading beads, drawing trail), (2) aiming and catching (catching beanbags, throwing beanbag onto mat) and (3) static and dynamic balance (one-leg balance, walking with heels raised, jumping on mats). Description and modality of measurements are described in the instructions accompanying the MABC-2 kit. The MABC has a minimum test–retest reliability at any age of 0.75 and an interrater reliability of 0.70 [47,48]. Low levels of agreement are found between the MABC and BOTMP [49] in the identification of children with motor difficulties (<80%), as MABC identified more children with motor difficulties compared to the latter [50,51]. A comparison of convergent validity between the MABC and the Peabody Developmental Motor Scales (PDMS-2) showed a correlation of 0.76 between the total score of the two tests, but a low agreement between the ability to identify motor impairment [52].

#### 2.5.3. Playground-Specific Tests

Two tasks, balancing on an elastic platform and balancing on a beam, were developed as a part of the project to assess aspects of dynamic balance. Similar test items also appear in the Körperkoordinationstest für Kinder: KTK [53].

#### 2.5.4. Balance on Elastic Platforms Task

Each elastic platform consists of a wooden disc (diameter: 54 cm) supported at 43 cm in height by a metal spring that makes it very unstable (Legnolandia, Italy, Jumpy, cod. 011107). The circuit consists of 6 platforms separated by gaps of 60 cm. The child begins with the feet parallel near the first platform; at the start signal, the child walks on the first platform and then jumps from platform to platform to the end of the circuit. The time is stopped when the participants jump down from the last platform. Time in seconds needed to complete the circuit and number of errors (every time the child goes down of the platform) are recorded.

#### 2.5.5. Balance on Beam Task

The balance beam consists of a 300 cm-long beam, 20 cm in diameter and about 10 cm high (Legnolandia, Italy cod 011065). The child begins with the feet parallel 10 cm from the beam. At a start signal, the participant goes up and walks as fast as possible on the beam. The time is stopped when the participants arrive at the end of the beam. Time of execution and number of errors (every time the child goes down from the beam) are recorded.

Assessments of motor skills were performed individually in a quiet room in the kindergarten or at the playground. Each test item was explained and demonstrated, and each child could familiarize themselves with the tests with a trial before measurements were taken. Participants were given verbal encouragement and support throughout the testing procedure. Playground-specific tests were performed individually during school visits at the playground occurring before and after the ten visits.

### 2.6. Statistical Analysis

Kolmogorov–Smirnov tests, histograms and Q-Q plots were applied to examine normality assumptions of the variables’ statistical distributions. Summarized scores were computed separately for TMC, MABC-II and playground tests, respectively. For TMC, in which all scores are recorded in seconds, the summarized score was simply the summed performance across the four tasks. For MABC-II and playground tasks, which consist of different scoring procedures for different tasks, each individual score was normalized by dividing peak performance in the current sample by individual performance multiplied by 100. This procedure generated a score from 0 to 100 for everyone for each task. These scores were thereafter summarized and divided by number of tasks in MABC (8) and playground tests (4), respectively. A 3 (study group) and X 2 (gender) ANCOVAs were conducted on post-test scores for each of the test items from TMC, MABC-II and playground tests, with pre-test scores as covariates. Post hoc pairwise multiple comparisons were conducted with the alpha Bonferroni corrected, and the partial eta-squared (*η*^2^*_p_*) was applied as a measure of effect size. Predictive Analytics Software (PASW, IBM, US; previously SPSS) Version 25.0 was used for all statistical procedures with *p* < 0.05 as statistical significance criterion.

## 3. Results

### 3.1. Test of Motor Competence (TMC)

Descriptive statistics for the various tasks in the TMC are in Table 1. Based upon ANCOVAs for post-test scores adjusted for pretest scores, results indicate no study group (control, partly structured activity, free play) x gender interaction effect on any of the measures from TMC [*F* (1, 8) ≤ 1.57, *p* ≥ 0.21, *η*^2^*_p_* ≤ 0.03] and no significant main effect for gender [*F* (1, 8) ≤ 0.78, *p* ≥ 0.38, *η*^2^*_p_* ≤ 0.007] in any of the TMC tests. Furthermore, a significant main effect of study group was found for all TMC measures [*F* (1, 8) ≥ 3.74, *p* ≤ 0.03, *η*^2^*_p_* = 0.07]. Further examination of post hoc Bonferroni-corrected comparisons, however, indicated no significant between-group differences in any of the TMC post-test scores (*p* ≥ 0.68). 

#### Movement Assessment Battery for Children (MABC-2)

Descriptive statistics for MABC-2 are depicted in Table 2. ANCOVAs for adjusted post-test scores (pre-test scores as covariates) indicated no study group (control, partly structured activity, free play) x gender interaction effect on any of the MABC-2 measures [*F* (1, 8) ≤ 2.73, *p* ≥ 0.07, *η*^2^*_p_* ≤ 0.05]. Further, no significant main effect was found for gender [*F* (1, 8) ≤ 1.78, *p* ≥ 0.18, *η*^2^*_p_* ≤ 0.04] in any of the MABC-2 tests, albeit a significant main effect of study group was found for the summarized score, throwing, and balance [*F* (1, 14) ≥ 4.25, *p* ≤ 0.2, *η*^2^*_p_* = 0.07]. Further examination of post hoc Bonferroni-corrected comparisons, however, indicated that the main effect of study group on post-test scores was due to a significant difference in post-test scores between the control group and the other two study groups (*p* < 0.001), as the control group had poorer scores on the post-test compared to the pretest scores, and no significant change in MABC-II measures was observed in the other two groups (*p* > 0.05).

### 3.2. Playground-Specific Assessments

Pretest–post-test values for the playground tasks can be found in Table 3. ANCOVAs on post-test scores adjusted for pre-test scores indicated no study group (control, partly structured activity, free play) x gender interaction effect on any of the playground measures [*F* (1, 8) ≤ 1.20, *p* ≥ 0.31, *η*^2^*_p_* ≤ 0.03]. Further, no significant main effect was found for gender [*F* (1, 8) ≤ 1.43, *p* ≥ 0.24, *η*^2^*_p_* ≤ 0.02] in any of the playground measures, and a significant main effect of study group [F (1, 14) ≥ 3.96, *p* ≤ 0.02, *η*^2^*_p_* = 0.09] was found for summarized playground and elastic platform scores. Post hoc Bonferroni-corrected comparisons indicated a significant difference in post-test summarized playground and elastic platform scores between the partly structured activity group compared to free play and control groups (*p* < 0.001). 

## 4. Discussion

The main aim of the study was to compare how the organization of a series of movement sessions as a partly structured activity or free play influenced development of motor competence in 4–5-year-old children. The results indicate that a significant improvement occurred only in the partly structured activity group and was limited to the summarized playground and elastic platform scores. These findings may indicate that development of skills was specific for those practiced in the playground, i.e., balance. It should be noted that the children of the partly structured activity trained 10 times for 10 min/week with the same equipment that was used for testing. The children in this group were practicing with a set of specific equipment which had a direct impact on that task measured; interestingly, the training had no effects on other tasks in the balance domain, such as heel-to-toe walking or standing on one leg. This result is in line with research on task-specific intervention in children with developmental coordination disorder (DCD), which is recommended to be task- and context-specific (what is to be learned, and for which circumstances) [25,54].

In the present study, the goal of the balance beam and of the elastic balance platform activities was to walk over to the end of the path as quickly as possible and without falling. The children in the partly structured group were provided with scaffolding by the trained educator if requested, e.g., instructions on how to use the instruments and general encouragement for the various aspects and challenges of the playground activities. Support, encouragement and guidance from a trained educator could maintain children’s attention on the goal of the activity over time. They were also given feedback or could use the adult’s arm to hold on to when balancing on the play equipment. This is in line with the approach of “deliberate preparation” [18,19]. As some activities in the playground, in the balance area, were in the “zone of proximal development” [55], during the execution of the activities and motor learning process, the children were supported by individualized feedback and scaffolding, when necessary. They were helped by the educator, who, for example, offered them their arm so that they could hold on in difficult moments. The zone of proximal developmental represents the level of difficulty that the child is unable to overcome on his own but which he overcomes with the support of an adult or an experienced peer. Tortella and Fumagalli [56] highlighted that children abandon the task after several attempts if the task is too difficult; on the contrary, they persist in their trials if supported by a specialized adult. As a consequence, they increase the time they spend practicing the difficult task and improve their performance and the outcome of the task. 

No significant changes related to the skills measured in the TMC or the MABC-2 between groups after ten weeks of movement activities in the playground were found. This could be explained by too little experience (i.e., time spent on task, such as duration and frequency) to affect general motor skill development measured by these test batteries, and/or to a dearth of task salience of the activities meant to prime progression in motor competence as measured by the TMC and MABC-2 [24]. For example, there was no loose equipment in the playground, while the MABC-2 requires throwing, catching and posting coins. Since the structured activity program in the playground focused on gross motor skills, the result suggests that transfer of motor competences from gross to fine motor domains is limited [57,58]). It was interesting to note during the data collection at the playground that the children of the free-play group usually avoided playing on the elastic balance platforms and the balance beam; as a consequence, they accumulated little or no experience with tasks based on the use of this equipment. 

The general lack of a tendency to improve after the intervention (as measured by TMC and MABC2 test batteries) could also be due to individual variation and variability typical of motor development in early childhood, e.g., the children have a wide repertoire of motor strategies (variation) and a high ability to vary the motor behavior to fit the situation (variability) [59]. According to Henderson and Sugden [7], the first phases in the motor learning process are characterized by attempts to understand what the task requires, its purpose, how it should be carried out and what strategies to use (i.e., children need to understand the skill). In this early period of motor development, learning is experimental, and the child needs to discover through rehearsal and experience [7]. Hence, the performance and outcome of the movement are not qualitatively stable yet [60] and may underlie the different outcomes obtained with the different test batteries. 

Overall, evidence related to frequency and/or duration and contextual information regarding the effect of the type, location, and organization of physical activity on improving preschool children’s motor skills is still unclear. Nevertheless, evidence regarding the effectiveness of physical activity interventions in motor skill development in preschoolers is strong [61]. Early child education and care (ECEC) institutions are central to children’s development, learning and health. In this context, the physical environment is an integral part of the learning environment in ECEC institutions and is of importance for children’s well-being, experiences and learning [62]. The physical environment may influence children’s possibilities for play and exploration, and young children should be provided more outdoor playtime in environments which are conducive to increased physical activity during childcare hours [63]. Several attributes of the outdoor environment seem to impact children’s physical activity levels and motor development, including paths, portable and fixed play equipment, open spaces and natural elements [64]. The different characteristics of these attributes enable activities and motor skills, and different terrain, materials, surfaces, spaces and areas in the outdoor environment are perceived as affordances and offer, invite and inspire the child to move and be active [65,66]. Based on the existing evidence, it is important to ensure preschoolers’ access to activities and environments that promote physically active play and motor activities and that reduce sedentary behavior. Children’s motor competence and physical activity behavior should be encouraged, as they may serve as positive and sustainable trajectories of health behavior and lead to positive long-term health outcomes [67]. Habits and patterns of behavior are established from an early age, and preschools should enable children to discover the joy of movement.

We acknowledge that this study may have some limitations as we do not know what kind of other physical activities the children participated in during the day, or on the weekends. Moreover, the participants were not chosen by cluster randomization, and no intracluster correlations (ICCs) were calculated due to the mode’s sample size. The strength of this study is that both groups had access to the same playground setting, thus allowing adequate comparison between groups. Additionally, the pragmatic real-world nature of the trial is a strength.

Physical activity and movement behavior are complex phenomena that are challenging to assess in young children. However, Truelove et al. [16] argue that a combination of accelerometers with an observational component could give a more complete picture in playground settings and enhance the evidence base related to young children’s participation in physical activity. Both information about intensity (via accelerometry) and contextual information regarding the type and location of activity behavior via observational tools could be essential. Further, developing valid and reliable motor competence assessments to understand current levels of motor competence in preschool children is also imperative.

## 5. Conclusions

The results indicate no significant differences in motor competence as measured by the TMC or the MABC-2 between groups. Only a significant improvement in performance in the summarized playground and elastic platform scores was observed in the partly structured activity group compared to the free-play and control groups. The frequency and/or duration as well as what contextual information of physical activity is needed to improve preschool children’s motor skills still needs to be explored. However, outdoor playtime may give possibilities for play, exploration and increased physical activity during childcare hours. In this context, it is important to ensure preschoolers’ access to activities and environments that promote physically active play and thus reduce sedentary behavior.

## Figures and Tables

**Table 1:** Results (means, SD) from Test of Motor Competence (TMC) at pretest and posttest across study groups.

	Study Group					
	Control		Structured Activity		Free Play	
Variable	Pretest	Posttest	Pretest	Posttest	Pretest	Posttest
Placing bricks (s)	83.54 (12.17)	92.91 (12.93)	93.94 (18.86)	98.93 (21.77)	88.51 (15.15)	87.84 (17.65)
Building bricks (s)	23.32 (5.27)	28.70 (9.54)	25.42 (7.94)	29.31 (9.28)	24.07 (5.77)	27 (6.72)
Heel-to-Toe walking (s)	34.61 (12.67)	38.27 (9.55)	39.45 (15.94)	38.85 (9.18)	41.09 (11.77)	40.93 (11.64)
Walking/running in slopes (s)	8.59 (2.08)	9.07 (2.51)	11.41 (3.64)	9.08 (2.73)	12.47 (3.71)	8.33 (1.71)
Sumscore (s)	125.11 (46.51)	154.33 (36.11)	149.66 (44.94)	152.72 (44.68)	149.88 (40.42)	153.01 (27.32)

**Table 2:** Results (means, SD) from Movement Assessment Battery for Children-II (MABC-II) at pretest and posttest across study groups.

	Study Group					
	Control		Structured Activity		Free Play	
Variable	Pretest	Posttest	Pretest	Posttest	Pretest	Posttest
Posting Coins (s)	50.30 (7.34)	46.81 (5.73)	54.06 (9.47)	48.35 (10.70)	51.70 (11.45)	46.34 (7.16)
Threading beads (s)	70.15 (15.27)	66.07 (14.51)	80.98 (28.29)	72.37 (31.86)	74.56 (27.00)	68.58 (26.45)
Drawing Trail errors (n)	1.45 (1.30)	1.97 (1.59)	2.02 (2.82)	2.27 (2.71)	1.92 (1.92)	1.82 (2.22)
Catching beanbags (n)	5.54 (2.73)	8.06 (1.53)	5.86 (2.77)	7.66 (2.15)	5.78 (3.16)	7.49 (1.98)
Throwing beanbags (n)	4.00 (4.58)	4.57 (1.84)	2.68 (2.02)	4.02 (2.18)	2.90 (1.77)	2.81 (1.93)
One leg balance (s)	23.85 (14.94)	30.27 (15.50)	27.00 (22.88)	17.54 (8.77)	29.13 (19.34)	18.11 (16.68)
Walking heels raised (n)	10.72 (5.77)	11.83 (4.21)	8.54 (6.00)	6.79 (5.57)	9.10 (5.92)	8.71 (5.80)
Jumping on mats (n)	2.59 (1.81)	2.00 (1.72)	1.52 (1.57)	1.93 (1.82)	1.56 (1.53)	2.15 (1.95)
Sumscore	45.42 (14.67)	55.95 (7.35)	45.24 (15.21)	46.73 (15.07)	47.81 (15.49)	49.73 (14.26)

**Table 3:** Results (means, SD) from playground-specific tests at pretest and posttest across study groups.

	Study Group					
	Control		Structured Activity		Free Play	
Variable	Pretest	Posttest	Pretest	Posttest	Pretest	Posttest
Elastic (s)	27.26 (12.70)	20.28 (11.72)	21.79 (11.11)	12.41 (5.93)	28.64 (17.92)	14.42 (11.00)
Elastic (n errors)	1.16 (1.65)	1.55 (2.06)	0.98 (1.75)	0.11 (0.49)	1.79 (2.14)	0.55 (1.39)
Beams (s)	22.79 (14.14)	14.18 (7.84)	24.97 (18.86)	13.03 (11.20)	23.30 (15.79)	10.44 (5.28)
Beams (n errors)	3.91 (3.58)	2.17 (2.14)	3.25 (3.06)	1.84 (2.92)	4.45 (3.87)	1.47 (1.75)
Sumscore	46.48 (14.23)	48.21 (10.05)	47.40 (13.05)	53.90 (1.93)	44.39 (13.68)	47.39 (12.02)

## Data Availability

The data that support the findings of this study are available from the corresponding author, [MH], upon reasonable request.

## References

[1] Bardid F., Rudd J., Elenoir M., Polman R., Barnett L.M. (2015). Cross-cultural comparison of motor competence in children from Australia and Belgium. Front. Psychol..

[2] Brian A., Bardid F., Barnett L.M., Deconinck F.J., Lenoir M., Goodway J.D. (2018). Actual and Perceived Motor Competence Levels of Belgian and United States Preschool Children. J. Mot. Learn. Dev..

[3] Brian A., Pennell A., Taunton S., Starrett A., Howard-Shaughnessy C., Goodway J.D., Wadsworth D., Rudisill M., Stodden D. (2019). Motor Competence Levels and Developmental Delay in Early Childhood: A Multicenter Cross-Sectional Study Conducted in the USA. Sports Med..

[4] Andersen E., Borch-Jenssen J., Øvreås S., Ellingsen H., Jørgensen K.A., Moser T. (2017). Objectively measured physical activity level and sedentary behavior in Norwegian children during a week in preschool. Prev. Med. Rep..

[5] Ellis Y.G., Cliff D.P., Janssen X., Jones R.A., Reilly J.J., Okely A.D. (2017). Sedentary time, physical activity and compliance with IOM recommendations in young children at childcare. Prev. Med. Rep..

[6] Burton A.W., Rodgerson R.W. (2001). New Perspectives on the Assessment of Movement Skills and Motor Abilities. Adapt. Phys. Act. Q..

[7] Henderson S.E., Sugden D.A., Barnett A. (2007). Movement Assessment Battery for Children.

[8] Cattuzzo M.T., Henrique R.D.S., Ré A., de Oliveira I.S., Melo B.M., Moura M.D.S., de Araújo R.C., Stodden D. (2016). Motor competence and health related physical fitness in youth: A systematic review. J. Sci. Med. Sport.

[9] Lima R.A., Pfeiffer K., Larsen L.R., Bugge A., Møller N.C., Anderson L.B., Stodden D. (2017). Physical Activity and Motor Competence Present a Positive Reciprocal Longitudinal Relationship Across Childhood and Early Adolescence. J. Phys. Act. Health.

[10] Vedul-Kjelsås V., Sigmundsson H., Stensdotter A.K., Haga M. (2012). The relationship between motor competence, physical fitness and self-perception in children. Child Care Health Dev..

[11] Timler A., McIntyre F., Rose E., Hands B. (2019). Exploring the influence of self-perceptions on the relationship between motor competence and identity in adolescents. PLoS ONE.

[12] Jefferies P., Ungar M., Aubertin P., Kriellaars D. (2019). Physical Literacy and Resilience in Children and Youth. Front. Public Health.

[13] Sigmundsson H., Haga M. (2016). Motor competence is associated with physical fitness in four to six-year-old pre-school children. Eur. Early Child. Educ. Res. J..

[14] Stodden D.F., Goodway J.D., Langendorfer S.J., Roberton M.A., Rudisill M.E., Garcia C., Garcia L.E. (2008). A developmental perspective on the role of motor skill competence in physical activity: An emergent relationship. Quest.

[15] Hulteen R.M., Morgan P.J., Barnett L.M., Stodden D.F., Lubans D.R. (2018). Development of Foundational Movement Skills: A Conceptual Model for Physical Activity Across the Lifespan. Sports Med..

[16] Truelove S., Vanderloo L.M., Tucker P. (2017). Defining and Measuring Active Play Among Young Children: A Systematic Review. J. Phys. Act. Health.

[17] Lubans D.R., Morgan P.J., Cliff D.P., Barnett L.M., Okely A.D. (2010). Fundamental movement skills in children and adolescents: Review of associated health benefits. Sports Med..

[18] MacNamara A., Collins D., Giblin S. (2015). Just let them play? Deliberate preparation as the most appropriate foundation for lifelong physical activity. Front. Psychol..

[19] Giblin S., Collins D., MacNamara A., Kiely J. (2014). “Deliberate Preparation” as an Evidence-Based Focus for Primary Physical Education. Quest.

[20] WHO (2019). Guidelines on Physical Activity, Sedentary Behaviour and Sleep for Children under 5 Years of Age.

[21] Newell K.M., Wade M.G., Whiting H.T.A. (1986). Constraints on the Development of Coordination. Motor Development in Children: Aspects of Coordination and Control.

[22] Adolph K.E., Hoch J.E. (2019). Motor Development: Embodied, Embedded, Enculturated, and Enabling. Annu. Rev. Psychol..

[23] Yu J.J., Burnett A.F., Sit C.H. (2018). Motor Skill Interventions in Children with Developmental Coordination Disorder: A Systematic Review and Meta-Analysis. Arch. Phys. Med. Rehabil..

[24] Kleim J.A., Jones T.A. (2008). Principles of experience-dependent neural plasticity: Implications for rehabilitation after brain damage. J. Speech Lang. Hear. Res..

[25] Smits-Engelsman B., Vincon S., Blank R., Quadrado V.H., Polatajko H., Wilson P.H. (2018). Evaluating the evidence for motor-based interventions in developmental coordination disorder: A systematic review and meta-analysis. Res. Dev. Disabil..

[26] Okely A.D., Booth M., Patterson J.W. (2001). Relationship of Physical Activity to Fundamental Movement Skills among Adolescents. Med. Sci. Sports Exerc..

[27] Wrotniak B.H., Epstein L.H., Dorn J.M., Jones K.E., Kondilis V.A. (2006). The relationship between motor proficiency and physical activity in children. Pediatrics.

[28] Palma M.S., Pereira B.O., Valentini N.C. (2014). Guided play and free play in an enriched environment: Impact on motor development. Motriz.

[29] Robinson L.E., Palmer K.K., Bub K.L. (2016). Effect of the children’s health activity Motor Program on Motor skills and self-regulation in head start Preschoolers: An efficacy Trial. Front. Public Health.

[30] Robinson L.E., Veldman S.L., Palmer K.K., Okely A.D. (2017). A Ball Skills Intervention in Preschoolers: The CHAMP Randomized Controlled Trial. Med. Sci. Sports Exerc..

[31] Tortella P., Haga M., Lorås H., Sigmundsson H., Fumagalli G. (2016). Motor skill development in Italian pre-school children induced by structured activities in a specific playground. PLoS ONE.

[32] Veldman S.L., Jones R.A., Okely A.D. (2016). Efficacy of gross motor skill interventions in young children: An updated systematic review. BMJ Open Sport Exerc. Med..

[33] Johnstone A., Hughes A.R., Martin A., Reilly J.J. (2018). Utilising active play interventions to promote physical activity and improve fundamental movement skills in children: A systematic review and meta-analysis. BMC Public Health.

[34] Palmer K.K., Matsuyama A.L., Robinson L.E. (2017). Impact of structured movement time on preschoolers’ physical activity engagement. Early Child. Educ. J..

[35] Pate R.R., Brown W.F., Pfeiffer K.A., Howie E.K., Saunders R.P., Addy C.L., Dowda M. (2016). An intervention to increase physical activity in children: A randomized controlled trial with 4-year-old in preschools. Am. J. Prev. Med..

[36] Tortella P., Haga M., Ingebrigtsen J.E., Sigmundsson H., Fumagalli G.F. (2019). Comparing free play and partly structured play in 4-5-years-old children in an outdoor playground. Front. Public Health.

[37] True L., Pfeiffer K.A., Dowda M., Williams H.G., Brown W.H., O’Neill J.R., Pate R.R. (2017). Motor competence and characteristics within the preschool environment. J. Sci. Med. Sport.

[38] Niemistö D., Finni T., Haapala E.A., Cantell M., Korhonen E., Sääkslahti A. (2019). Environmental Correlates of Motor Competence in Children—The Skilled Kids Study. Int. J. Environ. Res. Public Health.

[39] Sugiyama T., Okely A.D., Masters J.M., Moore G. (2012). Attributes of childcare centers and outdoor play areas associated with preschoolers’ physical activity and sedentary behavior. Environ. Behav..

[40] Adams J., Veitch J., Barnett L. (2018). Physical Activity and Fundamental Motor Skill Performance of 5–10-Year-Old Children in Three Different Playgrounds. Int. J. Environ. Res. Public Health.

[41] Buzzavo G., Dalt L.D., Durigon V., Fumagalli G., Maffeis C., Moghetti P., Romano M., Tortella P. (2011). Surroundings and Activities just Right for Growing Up Well Milano: Edizioni Libreria dello Sport. https://www.0246.it/wp-content/uploads/2018/06/book-eng-low.pdf.

[42] Sigmundsson H., Lorås H., Haga M. (2016). Assessment of motor competence across the life span: Aspects of reliability and validity of a new test battery. SAGE Open.

[43] Yoon D.Y., Scott K., Hill M.N., Levitt N.S., Lambert E.V. (2006). Review of three tests of motor proficiency in children. Percept. Mot. Ski..

[44] Rinne M.B., Pasanen M.E., Miilunpalo S.I., Oja P. (2001). Test-retest reproducibility and inter-rater reliability of a motor skill test battery for adults. Int. J. Sports Med..

[45] Rooks D.S., Kiel D.P., Parsons C., Hayes W.C. (1997). Self-paced resistance training and walking exercise in community-dwelling older adults: Effects on neuromotor performance. J. Gerontol. Ser. A Psychol. Sci. Soc. Sci..

[46] Johansson G., Jarnlo G.-B. (1991). Balance training in 70-year-old women. Physiother. Theory Pract..

[47] Henderson S.E., Sugden D.A. (1992). The Movement Assessment Battery for Children.

[48] Tan S.K., Parker H.E., Larkin D. (2001). Concurrent validity of motor tests used to identify children with motor impairment. Adapt. Phys. Act. Quart..

[49] Bruininks R.H. (1978). Bruininks-Oseretsky Test of Motor Proficiency: Examiners Manual Circle Pines.

[50] Crawford S.G., Wilson B.N., Dewey D. (2001). Identifying developmental coordination disorder: Consistency between tests. Phys. Occup. Ther. Pediatr..

[51] Slater L.M., Hillier S.L., Civetta L.R. (2010). The clinimetric properties of performance-based gross motor tests used for children with developmental coordination disorder: A systematic review. Pediatr. Phys. Ther..

[52] Van Waevelde H., Peersman W., Lenoir M., Smits Engelsman B.C.M. (2007). Convergent validity between two motor tests: Movement ABC and PDMS-2. Adapt. Phys. Act. Quart..

[53] Kiphard E.J., Schilling F. (2007). Körperkoordinationstest für Kinder 2, überarbeitete und ergänzte Aufgabe. Beltz test, Weinham. Med. Sci. Sports.

[54] Blank R., Barnett A.L., Cairney J., Green D., Kirby A., Polatajko H., Rosenblum S., Smits-Engelsman B., Sugden D., Wilson P. (2019). International clinical practice recommendations on the definition, diagnosis, assessment, intervention, and psychosocial aspects of developmental coordination disorder. Dev. Med. Child Neurol..

[55] Vygotsky L.S. (1962). Thought and Language.

[56] Tortella P., Fumagalli G. (2015). Activities in the zone of proximal development between the development of motor skills and school readiness: Studies in kindergartens. Eur. J. Res. Educ. Teach..

[57] Gallahue D.L., Ozmun J.C., Goodway J. (2012). Understanding Motor Development. Infants, Children, Adolescents, Adults.

[58] Sigmundsson H., Newell K.M., Polman R., Haga M. (2021). Exploration of the Specificity of Motor Skills Hypothesis in 7–8-Year-Old Primary School Children: Exploring the Relationship Between 12 Different Motor Skills from Two Different Motor Competence Test Batteries. Front. Psychol..

[59] Hadders-Algra M. (2010). Variation and Variability: Key Words in Human Motor Development. Phys. Ther..

[60] Sigmundsson H., Trana L., Polman R., Haga M. (2017). What is trained develops! theoretical perspective on skill learning. Sports.

[61] Zeng N., Ayyub M., Sun H., Wen X., Xiang P., Gao Z. (2017). Effects of physical activity on motor skills and cogni-tive development in early childhood: A systematic review. BioMed Res. Int..

[62] Ministry of Education and Research (2017). Framework Plan for the Content and Tasks of Kindergartens. https://www.udir.no/rammeplan.

[63] Truelove S., Bruijns B.A., Vanderloo L.M., O’Brien K.T., Johnson A.M., Tucker P. (2018). Physical activity and sedentary time during childcare outdoor play sessions: A systematic review and meta-analysis. Prev. Med..

[64] Sando O.J. (2019). The outdoor environment and children’s health: A multilevel approach. Int. J. Play..

[65] Haga M. (2021). Body and movement in early childhood: Spaces for movement-based play. J. Phys. Educ. Sport.

[66] Sääkslahti A., Niemistö D. (2021). Outdoor activities and motor development in 2–7-year-old boys and girls. J. Phys. Educ. Sport.

[67] Cairney J., Dudley D., Kwan M., Bulten R., Kriellaars D. (2019). Physical literacy, physical activity and health: Toward an evidence-informed conceptual model. Sports Med..

